# Spondyloarthritis Patients Suffer Increased Risk of Renal Complications Compared With General Population: A Retrospective Observational Study

**DOI:** 10.3389/fphar.2019.01073

**Published:** 2019-09-18

**Authors:** Min Xiao, Qing Lv, Yanli Zhang, Liudan Tu, Mingcan Yang, Zhiming Lin, Zetao Liao, Yutong Jiang, Xuqi Zheng, Xiaomin Li, Qiujing Wei, Shuangyan Cao, Jieruo Gu

**Affiliations:** Department of Rheumatology and Immunology, Third Affiliated Hospital of Sun Yat-sen University, Guangzhou, China

**Keywords:** spondyloarthritis, nephrolithiasis, renal insufficiency, retrospective study, risk factors

## Abstract

The objective of this study was to identify the prevalence and risk factors of renal complications of spondyloarthritis (SpA) patients, and to assess increased risks compared to general people. We conducted a retrospective study enrolled with consecutive SpA patients from an inpatient department and age, sex-matched general population (GP). The renal disorders investigated in this study contained decreased estimated glomerular filtration rate (eGFR), hematuria, proteinuria and nephrolithiasis. A total of 350 admitted SpA patients with complete medical records and 323 age and sex-matched GP were enrolled. Most SpA patients were male (n = 283, 80.9%) and the mean age was 31.61 ± 10.73 years old. Among 350 SpA patients, 29 (8.8%) suffered from hematuria, six (1.8%) suffered from proteinuria, one (0.3%) had decreased eGFR, and 27 (13.0%) presented with nephrolithiasis. The relative risk (RR) of nephrolithiasis in SpA compared to the GP was 2.24 (95% CI, 1.00-4.98), and the RR of renal insufficiency was 2.04 (95% CI, 1.11-3.77). In a univariate analysis, nephrolithiasis was significantly associated with age, age of onset, smoking, extra-articular manifestation and a bamboo spine. Renal insufficiency was significantly associated with age, peripheral manifestation, serum albumin, C-reactive protein and erythrocyte sedimentation rate. In a multivariable analysis, only extra-articular manifestation (OR = 8.43, 95% CI, 1.65-43.06, p = 0.010) and bamboo spine (OR = 3.47, 95% CI, 1.01-12.06, p = 0.049) remained significantly associated with nephrolithiasis. However, no variable was recognized as an independent risk factor for renal insufficiency. Renal complications are more common in SpA patients, with more than two-fold increased risk compared with GP. Extra-articular manifestation and bamboo spine are independent risk factors of renal disease in SpA patients.

## Introduction

Spondyloarthritis (SpA) is a chronic inflammatory disease characterized by spinal and sacroiliac joint involvement, of which ankylosing spondylitis (AS) is the main subtype (Sieper et al., 2009). As widely accepted, SpA is a male-predominant disease (male-female ratio is 2-3:1) (Lee et al., 2008; Kilic et al., 2017). SpA also causes peripheral rheumatological manifestations, e.g. peripheral arthritis, enthesitis and dactylitis, as well as SpA related extraarticular manifestations, e.g. uveitis, psoriasis and inflammatory bowel disease (Garg et al., 2014). As a chronic systemic inflammatory disease (Dulger et al., 2018), it has been reported that the risk of other organs being involved in SpA such as in osteoporosis (Wang et al., 2015), cardiovascular disease (Mathieu et al., 2015), gastrointestinal diseases (Molto et al., 2016) and renal diseases (Couderc et al., 2018), has increased. However, the accurate pathogenesis of these complications has not been thoroughly investigated.

Renal impairment with SpA mainly presents in several forms, including secondary renal amyloidosis, nonsteroidal anti-inflammatory drug (NSAID) nephropathy, glomerulonephritis and nephrolithiasis (Lee et al., 2013; Korkmaz et al., 2017). Recently there were several studies reporting the prevalence of renal complications with SpA (Kristensen et al., 2015; Barbouch et al., 2018; Couderc et al., 2018). However, as for the prevalence of renal abnormalities with SpA, the outcomes of researches vary largely. 5.2% of SpA patients exhibited renal dysfunction in the ASAS-COMOSPA study (Couderc et al., 2018), which recruited 3,984 patients with SpA from across 22 countries. In this study, renal dysfunction was defined as the estimated glomerular filtration rate (eGFR) < 60 ml/min/1.73 m^2^. However, a Swedish national register-based cohort study reported a much lower renal impairment prevalence (0.96%) in SpA, based on International Classification of Diseases (ICD) codes of renal insufficiency diagnosis (Kristensen et al., 2015). The discrepancy of prevalence derives from multiple factors, such as study design, recruited patients, different regions and diverse definitions of renal involvement. Moreover, only a few studies made comparisons of renal impairment between SpA and the general population (GP). A Canadian population-based study indicated that AS patients were at increased risk of chronic kidney disease (CKD) comorbidities compared to age, sex, and location matched controls (1.7% vs. 0.7%, OR = 2.4) (Haroon et al., 2015). Only CKD was studied in this research study, moreover, there is no study comparing the risks of renal impairment between SpA patients and the GP in China.

The objective of the present study was to access the prevalence of several types of renal complications in SpA patients and identify the predictive risk factors. Furthermore, we also assessed the increased risk of renal impairment among SpA patients compared to the GP.

## Materials and Methods

### Study Population

This retrospective study enrolled patients consecutively admitted to the inpatient department of the Third Affiliated Hospital of Sun Yat-sen University, from December 2015 to August 2018. All patients were diagnosed with SpA if the Assessment of SpondyloArthritis International Society (ASAS) classification criteria, a widely recognized criteria for SpA, was fulfilled (Rudwaleit et al., 2009; Zeidler and Amor, 2011). Other inclusion criteria were listed as follows: patients had to be over 16 years old and have complete relevant records including kidney function tests, urinalyses or renal ultrasonic examinations. Considering the relatively young onset of SpA, we set the age threshold to 16 years old instead of 18, to recruit more young patients and to explore their characteristics. We excluded subjects who developed signs of acute infection or those who have ever been diagnosed with a tumor. Patients suffering other rheumatic diseases or primary renal diseases were also excluded. The control group were age and sex-matched individuals who carried out regular health check-ups in our center and who had complete relevant records. This study was approved by the ethics committee of the Third Affiliated Hospital of Sun Yat-sen University ([2018]02-349-01) and conducted in compliance with the 1964 Helsinki Declaration.

### Data Collection

Clinical variables collected involved sex, age, age of onset, disease duration, body mass index (BMI), smoking, drinking, HLA-B27, peripheral manifestation and extra-articular manifestation. The severity of SpA was assessed according to the presence of bamboo spine. Treatment regimens concerning NSAIDs, disease-modifying anti-rheumatic drugs (DMARDs), tumor necrosis factor inhibitor (TNFi), glucocorticoid, calcium supplements and calciferol were collected. Laboratory indicators were also recorded such as aspartate transaminase (AST; U/L), alanine aminotransferase (ALT; U/L), total protein (TP; g/L), albumin (ALB; g/L), uric acid (UA; umol/L), immunoglobulin A (IgA; g/L), C-reactive protein (CRP; mg/L), and erythrocyte sedimentation rate (ESR; mm in the first hour).

The renal diseases investigated in this study included decreased renal function, abnormal urinalysis and nephrolithiasis, and the definitions were expounded in detail as follows: the status with values of estimated glomerular filtration rate (eGFR) level lower than 60 ml/min/1.73 m^2^ were defined as decreased renal function; eGFR was calculated according to the simplified Modification of Diet in Renal Disease (MDRD) formula (Levey et al., 2003); patients who developed hematuria or proteinuria of ≥grade1+ detected on at least two consecutive examinations were defined as having abnormal urinalysis; nephrolithiasis was diagnosed based on renal ultrasonic examination or previous nephrolithiasis medical history. We also checked the morphology of the urinary red cells of hematuria patients who underwent urinary sediment analysis. Dysmorphic erythrocytes more than 80% indicated glomerular origin; dysmorphic erythrocytes ranged from 20% to 80% indicating mixed hematuria, and lower than 20% suggested non-glomerular hematuria (Fairley and Birch, 1982). In addition, renal pathology of patients undergoing renal biopsy were also summarized. Given the different pathogenesis of nephrolithiasis and the other three types of renal involvement (hematuria, proteinuria and decreased eGFR) and to clarify the relevance of renal involvement and other factors more accurately, we divided renal involved patients into two groups, that is, the nephrolithiasis group and the renal insufficiency group. First, we compared the rate of hematuria, proteinuria and decreased eGFR in nephrolithiasis and non- nephrolithiasis group to evaluate the association between two types of renal impairment. We then analyzed the incidence rate and risk factors of two types of renal impairment, respectively.

### Statistical Analysis

According to several studies, the prevalence of renal disorders in SpA patients was 5.2%-21% (Barbouch et al., 2018; Couderc et al., 2018; Wu et al., 2018). According to the hypothesis that the prevalence of renal complications is 15% in SpA patients and about twice that in the GP, we estimated a sample size of 320 in the SpA group and 320 in the GP group, the study could achieve 90% power to detect a between-group difference. The test statistic used was the two-sided Z test with pooled variance and the significance level of the test was targeted at 0.05. Statistical analyses of data were performed using the IBM Statistical Package for Social Sciences (SPSS, version 20) software. Mean ± standard deviation (SD) was calculated for continuous variables, and frequency and percentage for categorical variables. The inter-group comparison was performed with the Student’s *t* test or Mann Whitney U test (Normal distribution of data was verified with the Kolmogorov-Smirnov test) for continuous variables and chi-squared tests for categorical variables (Fisher’s exact test where appropriate). Univariate logistic regression analysis was adopted to screen potential risk factors. The variable with a p-value less than 0.20 in the univariate analysis or clinical relevance was introduced into the multivariate logistic regression model. Co-linearity diagnostics were also performed on selected variables to determine the multicollinearity. Calibration of the final multivariate logistic model was assessed by the Hosmer-Lameshow test. Adjusted odds ratio (OR) and corresponding 95% confidence intervals (CI) were calculated and statistical significance was set at two‐sided *p* < 0.05.

## Results

### Demographic and Disease Characteristics of SpA Patients

A total of 350 admitted SpA patients whose medical records were complete and 323 age and sex-matched patients of the GP were enrolled from December 2015 to August 2018. Demographic and disease characteristics of SpA patients are shown in **Table 1**. Male patients (n = 283, 80.9%) made up the majority of enrolled SpA patients. The mean age was 31.61 ± 10.73 years old and the mean age of SpA onset was 24.72 ± 9.75 years old. The mean duration was 6.68 ± 6.06 years. 295 patients (91.6%) were HLA-B27 positive, while 48 patients (14.4%) had a positive family history. Peripheral manifestations were presented in 121 (34.6%) patients, extra-articular manifestations in 25 (7.1%), and 79 (26.9%) patients developed bamboo spine. All of the 350 included patients had serum creatinine data for eGFR calculation, 328 patients had carried out urinalysis, 207 of which had never carried out renal ultrasonic examination before. Among those patients, 29 patients (8.8%) suffered from hematuria, and six patients (1.8%) suffered from proteinuria. One patient (0.3%) presented a decreased eGFR, and 27 patients (13.0%) presented with nephrolithiasis.

**Table 1:** Demographic and disease characteristics of SpA patients.

Characteristics	All SpA (n = 350)
Male, n (%)	283(80.9)
Age, yrs, mean ± SD	31.61 ± 10.73
Age < 30 yrs, n (%)	172(49.1)
Age 30-45 yrs, n (%)	135(38.6)
Age ≥45 yrs, n (%)	42(12.3)
Age of onset, yrs, mean ± SD	24.72 ± 9.75
Duration, yrs, mean ± SD	6.68 ± 6.06
Smoke, n (%)	50(15.0)
Drink, n (%)	15(4.5)
Positive family history, n (%)	48(14.4)
B27(+), n (%)	295(91.6)
Peripheral manifestation, n (%)	121(34.6)
Extra-articular manifestation, n (%)	25(7.1)
Bamboo spine, n (%)	79(26.9)
Osteoporosis or Osteopenia, n (%)	67(51.9)
NSAIDS, n (%)	288(82.3)
DMARDS, n (%)	137(39.1)
TNFi, n (%)	165(47.1)
Glucocorticoid, n (%)	56(16.0)
Oral calcium, n (%)	79(23.8)
Calciferol, n (%)	110(33.2)
Manifestations of renal involvement	
Hematuria, n (%)	29(8.8)
Proteinuria, n (%)	6(1.8)
Decreased eGFR, n (%)	1(0.3)
Hematuria and proteinuria, n (%)	31(9.5)
Nephrolithiasis, n (%)	27(13.0)

SpA, spondyloarthritis; NSAIDs, nonsteroidal anti-inflammatory drugs; DMARDs, disease-modifying antirheumatic drugs; TNFi, tumor necrosis factor inhibitor; eGFR, estimated glomerular filtration rate.

As observed in **Table 2**, there were non-significant differences in renal insufficiency (18.5% vs 8.3%, *p* = 0.187) between the nephrolithiasis and non- nephrolithiasis groups, indicating that the involvement of nephrolithiasis (nephrolithiasis group) and the other three types (renal insufficiency group) was unassociated.

**Table 2:** Comparisons of renal insufficiency between nephrolithiasis and non- nephrolithiasis groups.

Variable	Nephrolithiasis	Non- nephrolithiasis	*p*
Renal insufficiency, n (%)	18.5	8.3	0.187^a^

a Chi-squared test was used for the significance of difference between two groups.

### Comparisons of Nephrolithiasis and Renal Insufficiency Between the SpA Cohort and the Matched GP

The mean age of the GP was 33.09 ± 9.97 years old and 75.2% was male which is similar to SpA patients (**Table 3**). Nephrolithiasis was detected by renal ultrasonic examination in 5.8% of the GP, and 4.3% suffered renal insufficiency, which is lower than the SpA cohort. The RR of nephrolithiasis in SpA patients compared to the GP was 2.24 (95% CI, 1.00-4.98), and the RR of renal insufficiency was 2.04 (95% CI, 1.11-3.77).

**Table 3:** Comparisons of nephrolithiasis and renal insufficiency between SpA cohort and matched general population.

	SpA	GP	*p*	RR (95%CI)
Male, n(%)	283 (80.9)	243 (75.2%)	0.078^a^	–
Age, yrs, mean ± SD	31.61 ± 10.73	33.09 ± 9.97	0.064^b^	–
Nephrolithiasis	13.0%	5.8%	0.040^a^	2.24 (1.00,4.98)
Renal insufficiency	8.9%	4.3%	0.019^a^	2.04 (1.11,3.77)

SpA, spondyloarthritis; GP, general population; RR, relative risk. ^a^Chi-squared test and ^b^Student’s t test were used for the significance of difference between two groups.

### Comparisons of Variables Between the Nephrolithiasis Group and the Non-Nephrolithiasis Group

As shown in **Table 4**, nephrolithiasis was significantly associated with age, age of onset, smoking, extra-articular manifestation and bamboo spine. Consistent with previous observations, elderly patients tended to develop nephrolithiasis more frequently (38.67 ± 11.67 vs 31.19 ± 11.69, *p* = 0.002). The age of onset was higher in patients with nephrolithiasis (30.00 ± 13.77 vs 24.73 ± 10.00, *p* = 0.016). Smoking tended to increase the risk of nephrolithiasis (33.3% vs 16.9%, *p* = 0.044). Compared with the non- nephrolithiasis group, patients with nephrolithiasis presented more extra-articular manifestation and bamboo spine (18.5% vs 5.0, *p* = 0.028 and 56.0% vs 20.3, *p* < 0.001). Unexpectedly, none of the treatment and biochemical indicators showed a significant association with nephrolithiasis.

**Table 4:** Comparisons of variables between the nephrolithiasis group with the non- nephrolithiasis group and the renal insufficiency group with the non- renal insufficiency group.

Variable	Nephrolithiasis (n = 27)	Non-nephrolithiasis (n = 180)	*p*	Renal insufficiency (n = 31)	Non-renal insufficiency (n = 319)	*p*
Demographic characteristics						
Male, n (%)	24(88.9)	138(76.7)	0.151^a^	27(87.1)	256(80.3)	0.355^a^
Age, mean ± SD	38.67 ± 11.67	31.19 ± 11.69	0.002* ^b^	35.87 ± 10.64	31.19 ± 10.66	0.020* ^b^
BMI, mean ± SD	22.82 ± 3.54	22.42 ± 3.59	0.606^b^	22.66 ± 3.20	22.55 ± 3.68	0.871^b^
Age of onset, mean ± SD	30.00 ± 13.77	24.73 ± 10.00	0.016* ^b^	27.23 ± 9.02	24.50 ± 9.79	0.138^b^
Duration, mean ± SD	8.22 ± 7.38	6.23 ± 6.51	0.147^b^	8.35 ± 6.78	6.52 ± 5.97	0.108^b^
Smoking, n (%)	9(33.3)	30(16.9)	0.044* ^c^	5(16.1)	45(14.9)	1.000^c^
Drinking, n (%)	0(0)	8(4.5)	0.552^c^	0(0)	15(5.0)	0.417^c^
Clinical manifestations						
Family history, n (%)	3(11.1)	25(14.1)	0.902^c^	8(25.8)	40(13.2)	0.102^c^
B27(+), n (%)	23(92.0)	156(89.7)	0.993^a^	24(82.8)	271(92.5)	0.146^a^
Peripheral manifestation, n (%)	13(48.1)	74(41.1)	0.490^a^	17(54.8)	104(32.6)	0.013* ^a^
Extra-articular manifestation, n (%)	5(18.5)	9(5.0)	0.028* ^a^	3(9.7)	22(6.9)	0.835^a^
Bamboo spine, n (%)	14(56.0)	30(20.3)	<0.001* ^a^	14(38.5)	30(26.4)	0.190^a^
Osteoporosis or Osteopenia, n (%)	7(70.0)	30(53.6)	0.536^a^	8(66.7)	59(50.4)	0.284^a^
Treatments						
NSAIDS, n (%)	22(81.5)	141(78.3)	0.709^a^	26(83.9)	262(82.1)	0.809^a^
DMARDS, n (%)	9(34.6)	70(39.1)	0.660^a^	15(48.4)	122(38.5)	0.282^a^
TNFi, n (%)	13(48.1)	61(33.9)	0.149^a^	16(51.6)	149(46.7)	0.602^a^
Glucocorticoid, n (%)	5(18.5)	32(17.8)	1.000^c^	6(19.4)	50(15.7)	0.782^c^
Oral calcium, n (%)	4(16.0)	34(20.0)	0.841^a^	8(27.6)	71(23.4)	0.616^a^
Calciferol, n (%)	6(24.0)	40(23.7)	0.971^a^	10(34.5)	100(33.1)	0.881^a^
Biochemical indicators						
AST (U/L), mean ± SD	20.93 ± 8.30	18.78 ± 6.78	0.139^b^	19.13 ± 5.61	19.75 ± 8.89	0.701^b^
ALT(U/L), mean ± SD	25.33 ± 20.44	21.89 ± 16.04	0.319^b^	22.45 ± 23.99	22.97 ± 19.36	0.890^b^
TP(g/L), mean ± SD	69.50 ± 4.86	70.34 ± 6.27	0.506^b^	70.71 ± 5.39	70.19 ± 7.19	0.695^b^
ALB(g/L), mean ± SD	40.91 ± 4.91	40.97 ± 4.56	0.950^b^	39.85 ± 5.04	41.97 ± 4.47	0.014* ^b^
UA(umol/L), mean ± SD	356.2 ± 139.3	365.2 ± 113.4	0.712^b^	348.6 ± 121.2	377.6 ± 109.9	0.175^b^
IgA(g/L), mean ± SD	3.34 ± 1.12	2.99 ± 1.19	0.252^b^	3.09 ± 0.99	3.00 ± 1.21	0.753^b^
CRP(mg/L), median (IQR)	14.4 (3.5-36.4)	15.4 (2.6-36.3)	0.694^d^	15.7 (3.8-50.2)	9.9 (2.1-27.5)	0.034* ^d^
ESR(mm/h), median (IQR)	17.0 (10.5-45.0)	21.0 (10.0-53.0)	0.586^d^	28.0 (10.0-63.0)	15.0 (6.0-38.5)	0.011* ^d^

*p < 0.05. BMI, body mass index; NSAIDs, m nonsteroidal anti-inflammatory drugs; DMARDs, disease-modifying antirheumatic drugs; TNFi, tumor necrosis factor inhibitor; AST, aspartate transaminase; ALT, alanine aminotransferase; TP, total protein; ALB, albumin; BUN, blood urea nitrogen; UA, uric acid; IgA, immunoglobulin A; CRP, C-reactive protein; ESR, erythrocyte sedimentation rate; IQR, interquartile range. ^a^Chi-squared test, ^b^Student’s t test, ^c^Fisher’s exact test and ^d^Mann Whitney U test were used for the significance of difference between two groups.

Bolded p values indicated significant difference.

### Comparisons of Variables Between the Renal Insufficiency Group and the Non-Renal Insufficiency Group

Patients with renal insufficiency were older and more frequently develop a peripheral manifestation (**Table 4**). In addition, the level of ALB, CRP and ESR were significantly different between patients with and without renal insufficiency. Patients with renal insufficiency had a lower level of ALB (39.85 ± 5.04 vs 41.97 ± 4.47, *p* = 0.014). In accordance with previous reports, the mean level of acute-phase inflammation indicators was also higher in the renal insufficiency group: CRP (15.7 (3.8-50.2) vs 9.9 (2.1-27.5), *p* = 0.034) and ESR (28.0 (10.0-63.0) vs 15.0 (6.0-38.5), *p* = 0.011). There was no significant difference in gender, smoking, age of onset, duration and treatment.

### Urinary Sediment Analysis and Renal Histopathology

Among patients with hematuria, nine patients underwent urine red blood cell morphology analysis using a phase contrast microscope. Of them, five patients (55.6%) had dysmorphic erythrocytes greater than 80% which indicated that the erythrocytes primarily came from the glomerulus. The rest of the patients had mixed hematuria with dysmorphic erythrocytes ranging from 20% to 80%. This indicated that these patients all went through glomerulus impairment. Fifteen patients underwent renal biopsy, 14 (93.3%) of whom were diagnosed with IgA nephropathy, and one patient was diagnosed with chronic glomerulonephritis with minor glomerular abnormalities. Demographic and clinical features of these patients are summarized in **Table 5** and **Table 6**.

**Table 5:** Urine red blood cell morphology and clinical features of nine SpA patients who underwent a urinary sediment analysis.

Patients	Age	Dysmorphic erythrocytes	Morphologically normal erythrocytes	Source
1	50≤age<55	5000	14000	mixed
2	45≤age<50	1000	0	glomerular
3	35≤age<40	1000	2000	mixed
4	40≤age<45	32000	13000	mixed
5	50≤age<55	163000	35000	glomerular
6	40≤age<45	275000	22000	glomerular
7	30≤age<35	11000	2000	glomerular
8	30≤age<35	17000	30000	mixed
9	25≤age<30	232000	21000	glomerular

**Table 6:** Renal pathology and clinical features of 15 SpA patients who underwent a renal biopsy.

Patients	Age	Renal clinical manifestation	Renal pathology
1	40≤age<45	Hematuria and proteinuria	IgA nephropathy
2	25≤age<30	Hematuria	IgA nephropathy
3	20≤age<25	Hematuria and proteinuria	IgA nephropathy
4	25≤age<30	Hematuria and proteinuria	IgA nephropathy
5	20≤age<25	Hematuria	IgA nephropathy
6	25≤age<30	Hematuria and proteinuria	IgA nephropathy
7	20≤age<25	Hematuria	IgA nephropathy
8	40≤age<45	Hematuria and proteinuria	IgA nephropathy
9	15≤age<20	Hematuria	chronic glomerulonephritis with minor glomerular abnormalities
10	25≤age<30	Hematuria and proteinuria	IgA nephropathy
11	20≤age<25	Hematuria and proteinuria	IgA nephropathy
12	25≤age<30	Hematuria and proteinuria	IgA nephropathy
13	45≤age<50	Hematuria	IgA nephropathy
14	45≤age<50	Hematuria	IgA nephropathy
15	25≤age<30	Hematuria	IgA nephropathy

### Univariate and Multivariate Logistic Regression Analysis

First, we conducted a univariate logistic regression analysis to identify potential risk factors, and variables with p-values less than 0.20 were introduced into a multivariate logistic regression analysis. As for nephrolithiasis, a total of nine variables such as gender, age, age of onset, duration, extra-articular manifestation, TNFi treatment, smoking, AST, and bamboo spine were included in the multivariable analysis (**Table 7**). Finally, only extra-articular manifestation (OR = 8.43, 95% CI, 1.65-43.06, *p* = 0.010) and bamboo spine (OR = 3.47, 95% CI, 1.01-12.06, *p* = 0.049) were significant. As observed in **Table 8**, 11 variables, including age, age of onset, duration et al., were selected into multivariable logistic regression analysis for renal insufficiency. However, no variable was significantly associated with renal insufficiency.

**Table 7:** Logistic regression analysis of risk factors for nephrolithiasis group.

Variables	Univariate analysis		Multivariate analysis	
	OR (95% CI)	*p*	OR (95% CI)	***p***
Gender	2.44 (0.70,8.49)	0.163	2.16 (0.46,10.21)	0.330
Age	1.05 (1.02,1.08)	**0.003**	1.06 (0.77,1.45)	0.721
Age of onset	1.04 (1.01,1.08)	**0.020**	0.97 (0.71,1.33)	0.864
Duration	1.04 (0.99,1.10)	0.152	0.95 (0.68,1.32)	0.740
Extra-articular manifestation	4.32 (1.33,14.05)	**0.015**	8.43 (1.65,43.06)	**0.010**
TNFi	1.81 (0.80,4.10)	0.153	2.28 (0.84,6.22)	0.108
Smoking	2.45 (1.01,5.97)	**0.049**	1.33 (0.42,4.20)	0.630
AST	1.04 (0.99,1.09)	0.146	1.05 (0.99,1.12)	0.118
Bamboo spine	5.01(2.07,12.14)	**< 0.001**	3.47 (1.01,12.06)	**0.049**

Hosmer-Lameshow test statistics: *χ*2: 12.858, degree of freedom: 8, p value = 0.117. The variables in multivariate regression models lack multicollinearity and had variance inﬂation value <2.

OR, odds ratio; ESR, erythrocyte sedimentation rate; TNFi, tumor necrosis factor inhibitor; AST, aspartate transaminase.

Bolded p values indicated significant difference.

**Table 8:** Logistic regression analysis of risk factors for the renal insufficiency group.

Variables	Univariate analysis		Multivariate analysis	
	OR (95% CI)	*p*	OR (95% CI)	*p*
Age	1.04 (1.01,1.07)	**0.022**	1.06 (0.78,1.46)	0.703
Age of onset	1.03 (0.99,1.06)	0.140	0.98 (0.71,1.34)	0.883
Duration	1.04 (0.99,1.10)	0.111	0.99 (0.71,1.38)	0.943
Peripheral manifestation	2.51 (1.19,5.29)	**0.015**	1.55 (0.55,4.36)	0.409
Family history	2.29 (0.96,5.46)	0.063	2.26 (0.77,6.62)	0.139
B27(+)	0.39 (0.14,1.21)	0.081	0.37 (0.09,1.58)	0.180
ALB	0.91 (0.84,0.98)	**0.015**	0.99 (0.88,1.12)	0.896
UA	1.00 (0.99,1.00)	0.174	0.99 (0.99,1.00)	0.449
CRP	1.01 (1.00,1.02)	**0.041**	1.01 (0.99,1.03)	0.333
ESR	1.02 (1.00,1.03)	**0.006**	1.00 (0.98,1.02)	0.997
Bamboo spine	1.80(0.78,4.16)	0.167	0.96 (0.31,2.98)	0.942

Hosmer-Lameshow test statistics: *χ*2: 12.392, degree of freedom: 8, p value = 0.135. The variables in multivariate regression models lack multicollinearity and had variance inﬂation value <2.

OR, odds ratio; ESR, erythrocyte sedimentation rate; ALB, albumin; UA, uric acid; CRP, C-reactive protein; ESR, erythrocyte sedimentation rate.

Bolded p values indicated significant difference

## Discussion

In the current study, the renal complications in SpA patients included hematuria, proteinuria, decreased eGFR and nephrolithiasis. Among the 350 SpA patients, 29 (8.8%) suffered from hematuria, six (1.8%) presented with proteinuria, the eGFR of one patient (0.3%) was decreased and 27 (13.0%) underwent nephrolithiasis. The RR of SpA patients who presented with renal insufficiency compared to the GP was 2.53 (95%CI, 2.19-2.93) and the RR of nephrolithiasis was 2.24 (95% CI, 1.00-4.98), respectively. These results imply that renal diseases are quite common in SpA patients and the risk of renal comorbidities among SpA patients was more than two times that of the GP.

Previous studies demonstrated that the prevalence of renal complication in SpA patients varied considerably. As for hematuria, it was exhibited in 41% (34/83) of patients with AS according to the cross-sectional study conducted in Turkey (Korkmaz et al., 2005). However, hematuria was reported to be only 1.2% in a large cohort of Brazilian SpA patients (Rodrigues et al., 2012). In regard to nephrolithiasis, self-reporting kidney stones was observed in 23 (29.11%) of the 79 SpA patients in an American retrospective cohort study (Canales et al., 2006), while 250 (2.9%) nephrolithiasis events were recorded in 8,572 AS patients in a prospective population-based nationwide Swedish cohort study (Jakobsen et al., 2014). The distinct results could be a result of several reasons, such as the different sample sizes, diverse races and different regions. In addition, diverse definitions of renal involvement also contributed to the discrepancy of incidence rate. In a Chinese retrospective study, Wu, et al. defined decreased renal function as an eGFR level lower than 60 ml/min/1.73 m^2^ with a prevalence of 0.1% (Wu et al., 2018). In contrast, an eGFR level lower than 90 ml/min/1.73 m^2^ was regarded as a reduced eGFR in a hospital-based Indian study, and the prevalence, 37.5%, was much higher (Saigal et al., 2017). The lower prevalence of a decreased eGFR (0.3%) in our study might be due to the relatively strict definition used. Moreover, the selection of patients in several studies based on ICD-coded diagnosis (Jakobsen et al., 2014; Levy et al., 2014; Haroon et al., 2015; Kristensen et al., 2015) would create a selection bias, because asymptomatic or mild SpA patients might be missed. The prevalence of renal complications depends on various factors, which should be considered when designing researches.

The risk of two types of renal disease in SpA patients was about twice that of the GP, namely nephrolithiasis (RR = 2.24) and renal insufficiency (RR = 2.53). Only a few publications compared the risk of renal disease between SpA patients and the GP. Previous studies also reported a higher risk of CKD among AS patients than the healthy control (1.7% vs 0.7%, *p* = 0.11 and 2.8% vs 1.8%, *p* < 0.05) (Levy et al., 2014; Haroon et al., 2015). In spite of the different renal impairment definitions, the results were consistent with our findings, which also increased the credibility of our findings. In our study, urinary sediment analysis showed that most hematuria was of glomerular origin and renal pathology showed IgA nephropathy was of the predominant pathology type. One study conducted in Brazil showed that of the 24 patients assessed for the presence of dysmorphic erythrocytes, eight (33.3%) had glomerular hematuria while the others had mixed hematuria (Azevedo et al., 2011). Other studies reported that IgA nephropathy accounted for 38% and 33.3% (Lee et al., 2013; Wu et al., 2018), which showed that IgA nephropathy was the most common renal pathological type. The conclusions were in line with ours, however, the proportions were different. These results indicated that an abnormality of renal function in SpA patients was a featured pf impaired glomerular.

Several variables were identified as potential risk factors of renal involvement in the univariate logistic regression analysis, such as gender, smoking, duration et al. However, in the multivariable analysis, only extra-articular manifestation (OR = 8.43, 95% CI, 1.65-43.06, *p* = 0.010) and bamboo spine (OR = 3.47, 95% CI, 1.01-12.06, *p* = 0.049) were recognized to be independent risk factors of renal diseases in SpA patients. Two studies reported that high BASDAI and BASFI scores increases the risk of nephrolithiasis (Lui et al., 2011; Fallahi et al., 2012). Contradictory conclusions were drawn in the BASMI score. Fallahi, et al. found that nephrolithiasis patients had a higher BASMI score, while no association was found with the BASMI score by Lui, et al. Two large cohort studies reported that biologic therapy was a risk factor for nephrolithiasis (Lui et al., 2011; Jakobsen et al., 2014), but our study showed that TNF-α treatment was not significantly different between the nephrolithiasis and non-nephrolithiasis group, though TNF-α was more frequently used in the nephrolithiasis group. Incel, et al. found the femur neck BMD in AS patients with urolithiasis was significantly lower than those without urolithiasis (Incel et al., 2006). Nevertheless, the rate of osteoporosis or osteopenia was not significantly different in our study. In the univariate and multivariate analysis, extra-articular manifestation (uveitis, psoriasis and inflammatory bowel disease) and radiological destruction (bamboo spine) was significantly associated with nephrolithiasis, which is in accordance with previous reports (Cansu et al., 2011; Jakobsen et al., 2014). AS patients were prone to suffer from bone destruction, abnormal bone formation, and a sub-clinical bowel disease. Additionally, calcium metabolic disorders and long-term use of NSAID also contributed to the formation of renal stones. In patients without a previous history of nephrolithiasis, AS patients still had an increased risk compared to the GP (Jakobsen et al., 2014), suggesting the causality between AS and pathogenesis of nephrolithiasis.

Several studies came to an agreement on the risk factor of age and gender in renal disorders. The risk of renal involvement increased with age (Kovacsovics-Bankowski et al., 2000; Korkmaz et al., 2005; Singh et al., 2007; Levy et al., 2014; Couderc et al., 2018), which was also in accordance with our findings that nephrolithiasis as well as renal insufficiency was associated with age in the univariate analysis. Males have been reported to be more likely to have renal involvement than females (Jakobsen et al., 2014; Levy et al., 2014), however, Wu, et al. arrived at the opposite conclusion (Wu et al., 2018). In our study, there was no significant association, even though nephrolithiasis and renal insufficiency were more common in males (**Table 4**, 88.9% vs 76.7%, *p* = 0.151 for nephrolithiasis and 87.1% vs 80.3%, *p* = 0.355 for renal insufficiency).

Previous studies concluded that the risk of kidney impairment increased in high disease activity or inflammatory indicators (CRP or ESR) (Omdal and Husby, 1987; Peeters et al., 1988; Samia et al., 2012; Donmez et al., 2013; Barbouch et al., 2018; Couderc et al., 2018; Wu et al., 2018). In our study, the level of CRP and ESR were significantly higher in patients with renal insufficiency, but there was no significance in the multivariable logistic regression analysis. SpA is a chronic inflammatory disease and chronic inflammation is postulated to play a major role in renal disease by promoting arteriosclerosis (Couderc et al., 2018) and vascular endothelial dysfunction (Azevedo and Pecoits-Filho, 2010). Advanced age and smoking could accelerate inflammation and increase renal involvement. In the intergroup analysis, ALB was lower in patients with renal insufficiency, but showed no significance in the multivariate analysis. Further research on the mechanisms involved are required to better understand the effect of inflammation on renal complications.

The primary limitation of this study is the retrospective design. In addition, we speculate that the complication rate is underestimated because the symptoms of the enrolled patients were more serious. Furthermore, due to the limited samples size, several variables in the multivariate models without significance require larger studies to validate our findings. To our knowledge, this is the first study that investigated the prevalence of nephrolithiasis in the Chinese population and which compared the prevalence of renal disease in SpA patients with the GP. Each patient had performed renal ultrasonic examination to confirm the presence of renal stones whether they presented with symptoms or not. Therefore, the obtained prevalence data were credible. Prospective studies with larger sample sizes, and more reasonable definitions of renal involvement need to be conducted to explore the risk compared with general individuals.

When renal involvement manifestations occur in SpA patients, the disease often progresses to a more serious or advanced stage. Therefore, SpA patients should avoid medications with kidney toxicity. Furthermore, we recommend several examinations as routine tests to screen for early renal involvement, including a kidney function test, urinalysis and renal ultrasonic examinations, especially for older patients, patients with high disease activity, and patients with extra-articular manifestation and bamboo spine. Patients that present with hematuria or proteinuria should undergo further examinations. Renal biopsy is required when necessary, especially for hematuria of glomerular origin. It is beneficial to guide further treatment and evaluate prognosis.

## Conclusions

Renal complications are more common in SpA patients, with a more than two-fold increased risk compared with the GP. Extra-articular manifestation and bamboo spine are the independent predictive factors of renal disease in SpA patients.

## Data Availability

All datasets generated for this study are included in the manuscript and/or the supplementary files.

## Ethics Statement

This study was carried out in accordance with the recommendations of the ethics committee of the Third Affiliated Hospital of Sun Yat-sen University ([2018]02-349-01) with written informed consent from all subjects. All subjects gave written informed consent in accordance with the Declaration of Helsinki. The protocol was approved by the ethics committee of the Third Affiliated Hospital of Sun Yat-sen University.

## Author Contributions

Study design: JG, MX, and QL. Data collection: MY, ZML, ZTL, YJ, XZ, XL, QW, and SC. Statistical analysis: MX, QL, YZ, and LT. Manuscript drafting: MX. Manuscript revision: QL, MY, ZML, and ZTL. Supervision: JG. Project administration: JG.

## Funding

This work was supported by the National Natural Science Foundation of China Grant (81571595), the National Natural Science Foundation of China Grant (81871294) and the Major program of Health Medical Collaborate Innovation of Guangzhou (201604020013).

## Conflict of Interest Statement

The authors declare that the research was conducted in the absence of any commercial or financial relationships that could be construed as a potential conflict of interest.
